# Testing a self-determination theory-based process model of physical activity behavior change in rheumatoid arthritis: results of a randomized controlled trial

**DOI:** 10.1093/tbm/ibaa022

**Published:** 2020-03-23

**Authors:** Sally A M Fenton, Jet JCS Veldhuijzen van Zanten, George S Metsios, Peter C Rouse, Chen-an Yu, Nikos Ntoumanis, George D Kitas, Joan L Duda

**Affiliations:** 1 School of Sport, Exercise and Rehabilitation Sciences, University of Birmingham, Birmingham, UK; 2 Department of Rheumatology, Russells Hall Hospital, Dudley Group NHS Foundation Trust, Dudley, UK; 3 Faculty of Education, Health and Wellbeing, University of Wolverhampton, West Midlands, UK; 4 Department for Health, University of Bath, Bath, UK; 5 Physical Activity and Well-Being Research Group, School of Psychology, Curtin University, Perth WA, Australia

**Keywords:** Rheumatoid arthritis, Self-determination theory, Autonomous motivation, Physical activity, Subjective vitality, Randomized controlled trial

## Abstract

Physical inactivity is prevalent in rheumatoid arthritis (RA) patients, increasing the risk of poor physical health and compromised well-being. Interventions are therefore required to support physical activity (PA) behavior change in this population. This study examined whether a self-determination theory (SDT) based exercise intervention for people with RA, increased autonomous motivation for PA and in turn, moderate-to-vigorous PA (MVPA) and subjective vitality RA patients (*n* = 115) were randomized to a 3-month SDT-based psychological intervention + RA-tailored exercise program (experimental group, *n* = 59) or a RA-tailored exercise program only (control group, *n* = 56). During the program, the SDT-based intervention group received one-on-one consultations with a PA advisor trained in delivering strategies to promote autonomous motivation for PA. Well-established questionnaires assessed autonomous and controlled motivation for PA, MVPA (min/week), and subjective vitality at baseline (T1) and 3 months (T2). Path analysis examined the hypothesized theoretical process model. The model demonstrated an excellent fit to the data (*n* = 70, *χ*^2^ (26) = 28.69, *p* = .33, comparative fit index = 0.99, root square mean error of approximation = 0.04). The intervention corresponded to higher autonomous motivation and lower controlled motivation for PA at T2, after controlling for T1 autonomous and controlled motivation. In turn, changes in autonomous motivation from T1 to T2 significantly positively predicted changes in MVPA and subjective vitality. Results suggest an SDT based psychological intervention comprising autonomy-supportive strategies for PA predicted greater reported autonomous reasons for PA in RA patients participating in a tailored 3-month exercise program. Increased autonomous motivation linked to increased engagement in MVPA and feelings of vitality in these patients.

## Teaser

An exercise programme tailored for people with Rheumatoid Arthritis, was successful at increasing physical activity and enhancing psychological well-being, when delivered in conjunction with psychological support for physical activity behaviour change. The psychological support aimed to encourage more autonomous motivation for physical activity participation.

**Implications**

**Practice:** The provision of autonomy-supportive strategies in the health care setting can support the adoption of more physically active lifestyles and improved psychological well-being among people living with rheumatoid arthritis, through promoting more autonomous reasons for engagement.

**Policy:** Policymakers who want to promote more active lifestyles as an avenue disease management should explore (i) how frontline health care professionals can be effectively trained in approaches to support autonomous motivation for physical activity or (ii) consider how specialized physical activity behavior change professionals can be integrated into health care pathways.

**Research:** Future research should confirm the potential value of autonomy-supportive interactions as a strategy to promote physical activity and improve psychological well-being among other clinical populations.

Rheumatoid arthritis (RA) is an autoimmune disease affecting approximately 0.2%–1% of the adult population worldwide [1, 2]. In RA, persistent synovial inflammation manifests as joint pain and swelling, leading to musculoskeletal deterioration and disability. In addition, chronic high-grade systemic inflammation incites other extra-articular disease manifestations, such as cardiovascular disease (CVD). The health burden of RA also means people living with this chronic disease are at significantly increased risk of compromised psychological well-being [3].

Physical activity (PA) is recommended for the management of RA outcomes [4]. Both prospective and experimental studies indicate higher levels of PA engagement to lead to improvements in inflammatory disease activity, physical function, CVD risk, and psychological health [4–7]. However, research suggests that people living with RA participate in very low levels of PA, especially at the intensity required to accrue health benefits—that is, moderate-to-vigorous PA (MVPA; ≥3 metabolic equivalents) [8, 9]. Common barriers to PA reported by RA patients are pain, fatigue, and fear of causing further joint damage [10], despite conclusive evidence that PA is safe in this population [11]. With this in mind, there is a great requirement for behavioral interventions that can better support people living with RA to participate in MVPA, in order to improve their physical and mental health.

For interventions to be effective, it is essential they target factors (i.e., determinants) that influence PA behavior [12, 13]. Psychological theories provide systematic frameworks to identify potential determinants and to also evaluate the cognitive and affective mechanisms through which these determinants may act to encourage behavioral change [13]. In this regard, self-determination theory (SDT) [14, 15] has been successfully applied to health behavior change interventions—including those targeting PA [16–18].

A fundamental concept of SDT is the assumption that the social environment is central to an individual’s quality of motivation to engage in a behavior [14]. Specifically, SDT proposes that social environments that support autonomy in regards to the target behavior (e.g., provide choices and options) will foster more autonomous motivation toward engagement [14, 19]. Autonomous motivation reflects intrinsic (e.g., fun, enjoyment) and/or personally identified reasons (e.g., perceived health benefits) for behavioral engagement and is often associated with adaptive cognitive, affective, and behavioral outcomes [19]. In contrast, more controlling environments undermine autonomous motivation, leading to controlled motivation whereby more introjected and external reasons guide engagement in a behavior (e.g., avoidance of shame and guilt, pressure from others). As a result, maladaptive cognitive, affective, and behavioral outcomes are likely to occur [14].

**Fig 1:**
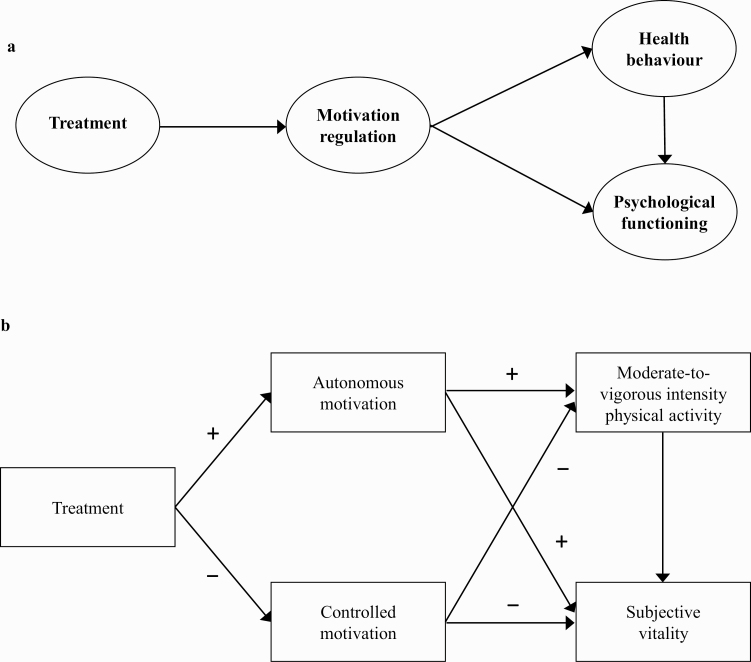
The SDT process model for health behavior change in intervention research (a) and hypothesized model (b). Arrows (b) indicate all associations tested in the hypothesized model. Symbols indicate the direction of the associations.

SDT advocates enhanced autonomous motivation as a psychological “process of change.” That is, strategies to promote autonomous motivation—such as the provision of autonomy—are assumed to relate to a subsequent positive change in targeted outcomes (e.g., PA, Fig. 1a) [17, 19]. In the context of PA intervention, examples of autonomy-supportive strategies that aim to facilitate more identified reasons for PA engagement include discussing participants’ exercise/PA history and eliciting and acknowledging previous experiences and emotions toward PA, sharing information regarding the benefits of PA that are likely to be salient to the individual, ensuring discussions are tailored so they are personally meaningful to the individual (e.g., providing rationale), and encouraging reflection on the links between PA and personally meaningful life goals or events.

Intervention studies conducted to promote PA in non-RA populations have employed such strategies and consistently demonstrate support for the role of autonomy support and ensuing autonomous motivation, as key psychological processes underlying positive PA behavior change [17, 18]. For example, a randomized controlled trial (RCT) demonstrated that primary care patients receiving an autonomy-supportive brief consultation from their General Practitioner, followed by 3-month SDT-based counseling from an exercise counselor, exhibited greater autonomous motivation and self-reported PA 3 months after the intervention [20]. Analysis of the psychological “processes of change” also revealed that mid-program levels of autonomous motivation toward PA mediated the increases in PA levels observed at the end of the program. Similarly, an SDT-based weight management program for women, in which health care providers were trained to deliver autonomy-supportive weight-loss sessions (focused promoting a healthy diet and PA), demonstrated increased autonomous motivation toward PA following the intervention [21]. In turn, enhanced autonomous motivation was found to encourage higher PA in the intervention group, supporting the change mechanisms hypothesized by SDT (Fig. 1a). However, for people with RA, the role of interventions centered on the delivery of autonomy-supportive strategies (e.g., within the health care setting) to promote autonomous motivation for, and engagement in, PA has not been examined.

In addition to the potential implications for PA, extant observational research has highlighted other positive concomitants of autonomous motivation, including psychological well-being [22]. For example, studies have revealed autonomy-supportive strategies and resulting autonomous motivation toward PA to associate with reduced prevalence of depressive symptoms and improved subjective vitality in people with RA [23], patients on exercise referral [24], and physically inactive adults [25].

Subjective vitality (e.g., feeling alive, full of energy and spirit) indicates the extent to which an individual is experiencing *eudaimonic* well-being, a positive indicator of optimal functioning and psychological health [26, 27]. Subjective vitality is considered to stem from an internal locus of causality, which is influenced by both physical and psychological factors [28]. For example, in the context of RA, an individual’s rheumatic pain (physical factor) may interfere with feelings of energy, but their degree of subjective vitality will influence their perception and perceived ability to cope with such adverse experiences. That is, a higher strength of vitality may act as a buffer to the physical challenges resulting from RA, representing energy that can be harnessed or regulated for purposive actions [26, 28]. Subjective vitality may therefore be an indicator of overall psychological functioning in people living with rheumatic disease. Indeed, a recent study in RA demonstrated significant positive associations between subjective vitality with quality of life and significant negative associations with fatigue, anxiety, and depression [26].

In summary, there are convincing theoretical and empirical reasons for employing SDT as a theoretical framework on which to develop and evaluate a PA intervention to be applied within the RA health care setting. Indeed, current evidence suggests that interventions targeting autonomous motivation for PA as a psychological “process of change” may have the potential for encouraging PA and improving psychological well-being in this patient group. In response, we developed a 3-month SDT-based exercise intervention for people living with RA, with the aim of promoting autonomous motivation for PA and, in turn, encouraging the adoption of MVPA and improving related psychological well-being. The SDT-based intervention was tested via an RCT, the protocol of which is detailed elsewhere [29]. Here, we test an SDT-based theoretical process model of change to examine the effects of the intervention on the targeted psychological process of change (i.e., autonomous motivation for PA) and related outcomes. Specifically, we tested a model to examine whether autonomy-supportive interactions with an SDT-trained behavior change counselor would positively predict changes in autonomous motivation toward PA and, in turn, encourage higher MVPA and increased subjective vitality, at the end of the 3-month exercise program (Fig. 1b).

## METHODS

Patients diagnosed with RA [30] were recruited from Rheumatology outpatient clinics at Russells Hall Hospital (Dudley, UK), between March 2010 and April 2014 (ISRCTN04121489). Exclusion criteria were recent joint surgery (preceding 6 months), fibromyalgia, and co-morbidity incompatible with exercise as per American College of Sports Medicine guidelines. Eligible participants provided informed consent and were randomized to the experimental or control group (stratified based on gender, by a third party [Cancer Clinical Trials Unit, University of Birmingham]). All randomized participants (both experimental and control) were invited to participate in a 3-month exercise program in a local gym, tailored for people living with RA. Participants in the experimental group also received an SDT-based psychological intervention to support autonomous motivation for engagement in PA [29].

### Exercise program (all participants)

The content of the exercise sessions was individualized and based on participants’ pre-intervention cardio-respiratory fitness (CRF) and physical abilities [7]. Full details of the exercise program are described elsewhere [7, 29]. In brief, participants were advised to complete 3 × 30 min exercise sessions per week (two in the gym + one at home, both at 60%–70% Heart Rate max). Participants completed at least one induction session in the gym until they felt confident to conduct exercises independently. Thereafter, participants exercised in the gym with instructors available to answer questions. To avoid treatment contamination, the experimental and control groups completed their programs in different gyms, with comparable facilities. Instructors at both gyms received training regarding exercise in RA, to ensure the exercise program was tailored for, and delivered in a manner sensitive to patient’s needs.

### SDT-based psychological intervention (experimental group only)

For patients randomized to the experimental group, the individualized and RA-tailored 3-month exercise program was supplemented by a psychological intervention grounded in SDT. Participants in the experimental group received four one-on-one consultations with a behavior change counselor, who was trained in the provision of strategies to promote more autonomous motivation for PA. Consultations were (i) one face-to-face consultation prior to initiating the exercise program (baseline), (ii) two telephone consultations from the behavior change counselor during the exercise program (1 and 2 months), and (iii) one face-to-face consultation upon completion of the program (3 months). During the initial consultation (baseline, 60 min duration), the behavior change counselor first elicited and acknowledged positive and negative experiences and emotions toward PA and sought to identify the patients’ knowledge of the benefits associated with PA for people living with RA. These discussions were geared toward benefits that were personally meaningful for the patients to foster more identified reasons for PA engagement (i.e., providing a meaningful rationale to promote autonomy). In this consultation, the behavior change counselor also encouraged the participant to reflect on the links between PA behavior and personally meaningful life goals or events, to support the internalization of reasons for engaging in PA. Decisional balance (weighing perceived pros and cons of participation in PA) and patient-centered goal setting were also addressed to encourage the adoption of PA.

The first telephone consultation (1 month, 10 min duration) involved the behavior change counselor supporting participants’ attempts to change their behavior and normalizing any failed attempts to be physically active. Counselors led patients in problem solving (identifying barriers to PA and formulating strategies/solutions to overcome them) to enhance patients’ self-efficacy for PA. Goals set during the initial face-to-face consultation were also revisited and revised by the patients in accordance with their perceived needs and capabilities. During the second telephone consultation (2 months, 10 min duration), behavior change counselors continued to encourage participants’ attempts to be physically active and again guided the patient in problem solving regarding overcoming barriers and goal setting for the last period of the program.

At the end of the exercise program (3 months), patients were to receive a second face-to-face consultation (30 min duration), during which discussions between the behavior change counselor and participant centered on recognizing and reinforcing: the internalization of participants’ PA participation, their feelings toward PA (through asking participants to verbalize these feelings), and plans to engage in PA in the future (including information about local PA opportunities and provisions). A detailed description of the content of the behavior change counselor consultations is also described elsewhere [29].

**Fig 2:**
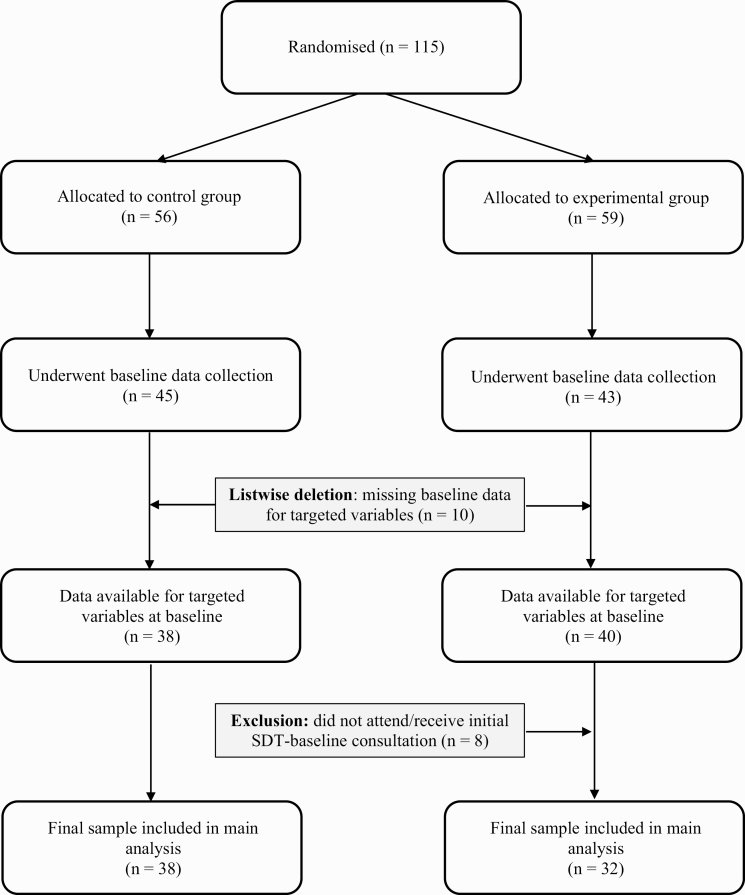
Flow diagram of participants in the study.

For example, during their initial consultation, the behavior change counselor discussed participants’ exercise history, encouraged identification of personally relevant benefits associated with PA (i.e., providing a rationale to promote autonomy), and encouraged reflection on the links between PA and personally meaningful life goals or events (facilitating identified reasons for engagement). For this study, we include only participants who received at least their first consultation with the behavior change counselor (Fig. 2). This first consultation (face-to-face) was the most extensive (i.e., 1 hr) and was geared toward supporting the adoption of PA. The content of the conversation between the behavior change counselor and patient in subsequent telephone (1 and 2 months) and face-to-face (3 months) consultations drew from and entailed recalling the points discussed in the initial exchange (e.g., to problem solve regarding barriers to PA participation and re-visit PA goals set) [29].

### Outcomes

All assessors were blinded to the intervention to which the participants were randomized. Assessments (full details have been previously published) [29] included measurement of RA characteristics (e.g., disease activity), physical health (e.g., blood pressure [BP], physical function), CRF, and questionnaires to assess autonomous and controlled motivation for PA, MVPA, and psychological well-being. In the present study, our focus was on responses to questionnaires administered at baseline (pre-randomization, T1) and at 3 months (end of the exercise program, T2).

#### Autonomous and controlled motivation for exercise engagement

Motivation regulations toward engagement in exercise were measured using the 19-item Behavioral Regulation in Exercise Questionnaire (BREQ-2) [31]. Following the stem “I participate in exercise because….,” this questionnaire requires participants to respond to items reflecting intrinsic motivation (e.g., “I exercise because it is fun”), identified regulation (e.g., “I value the benefits of exercise”), introjected regulation (“I feel guilty when I do not exercise”), external regulation (e.g., “I exercise because other people say I should”), and amotivation (e.g., “I do not see the point of exercising”). Participants rate their agreement with each statement on a five-point scale ranging from 0 (not true for me) to 4 (very true for me). Composite scores were computed to reflect autonomous motivation (intrinsic motivation + identified regulation) and controlled motivation (introjected regulation + external regulation) for inclusion in the hypothesized model. This is in accordance with past research examining the motivational processes underlying PA behavior change, including in RA [18, 23].

#### Subjective vitality

Positive mental health was assessed using the Subjective Vitality Scale (SVS) [28]. Following the stem… “During the past 3–4 weeks, in my everyday life….,” participants are asked to respond to five statements (e.g., “I feel alive and full of spirit”) on a five-point scale, ranging from 1 (strongly disagree) to 5 (strongly agree). The SVS demonstrated high internal reliability in this study (α = 0.93 [T1] and α = 0.94 [T2]) and has recently been validated for use in RA populations [26].

#### Moderate-to-vigorous physical activity

PA was self-reported by all participants using the International Physical Activity Questionnaire (IPAQ) [32]. The IPAQ measures the level of PA across four domains: leisure-time PA, domestic and gardening (yard) activities, work-related PA, and transport-related PA. In each domain, the duration (in minutes) and frequency (days) of PA including moderate and vigorous PA are reported, and weekly minutes spent in MVPA are calculated ([minutes × days moderate PA] + [minutes × days vigorous PA]). The IPAQ has been used previously to assess PA among patients with RA [33] and is recommended for use in intervention studies focused on promoting PA [34].

#### RA disease characteristics and physical health

To characterize the sample, we report data collected as part of the larger protocol, pertaining to participants’ RA disease duration, disease activity, functional ability and RA medication, and physical health (including CRF). Details of these assessments are provided elsewhere [29]. In brief, disease duration and current medication were self-reported and corroborated with the patient’s medical notes. RA disease activity was determined using the Disease Activity Score-28 (DAS-28) [35] and functional ability was measured using the Stanford Health Assessment Questionnaire Disability Index [36]. BP was assessed using an electronic sphygmomanometer (Datascope Accutor). Serological CVD risk factors were measured from fasting blood samples (e.g., C-reactive protein, high-density lipoprotein cholesterol), and the QRISK2 score was used to indicate global 10-year CVD risk [37]. CRF was assessed using a graded exercise tolerance test [29].

### Data reduction

Of the *n* = 115 participants recruited, *n* = 27 were excluded prior to baseline data collection due to a new diagnosis of heart rate irregularities (*n* = 4), high BP (*n* = 8), other medical condition (*n* = 6), loss of interest (*n* = 16), and lack of time (*n* = 3). Baseline data were therefore collected from *n* = 88 participants (experimental, *n* = 43; control, *n* = 45).

#### Participant exclusions

The flow of participants through the study is outlined in Fig. 2. In addition to aforementioned exclusions, a further *n* = 10 participants were removed from analyses due to failure to complete questionnaires for targeted variables at T1 (motivation regulations, MVPA, subjective vitality; experimental, *n* = 3 and control, *n* = 7), and *n* = 8 participants were excluded from the experimental group, who did not receive the initial SDT-based consultation (at baseline, following randomization). The final sample available for analysis was therefore *n* = 70 (experimental, *n* = 32; control, *n* = 38). Statistical tests revealed participants excluded on the basis of missing baseline questionnaire data (*n* = 10) or SDT consultation (*n* = 8) were not significantly different to the final *n* = 70 participants included, in terms of demographics, RA disease characteristics, and physical health (Table 1). However, included participants were significantly less likely to be on Anti-TNF therapy (*χ*^2^ (1) = 7.46, *p* = .01) and significantly more likely to be on Disease-Modifying Anti-Rheumatic Drugs (*χ*^2^ (1) = 4.83, *p* = .03) than those excluded.

**Table 1:** Participant characteristics; demographics, RA medication, disease characteristics, and physical health

Outcome measure	Total sample (*n* = 70)		Control group (*n* = 38)	Experimental group (*n* = 32)
	*M* ± *SD*	Range (min–max)	*M* ± *SD*	*M* ± *SD*
Demographics				
Age (years)	56.4 ± 12.3	24–80	55.5 ± 12.3	57.5 ±11.5
Female sex (*n*)	46 (66%)		27 (71%)	19 (59%)
Married/living with partner (*n*)	58 (83%)		31 (82%)	27 (84%)
RA medication				
DMARDs (*n*)	64 (91%)		34 (90%)	30 (94%)
Anti-TNF (*n*)	6 (9%)		2 (5%)	4 (13%)
NSAIDs (*n*)	21 (30%)		11 (29%)	10 (31%)
Corticosteroids	13 (19%)		8 (21%)	5 (16%)
Disease characteristics				
Disease duration (years)	7.8 ± 9.3	1–44	8.1 ± 10.0	7.5 ± 8.7
Disease activity (DAS-28)	3.0 ± 1.3	0.6–5.9	3.1 ± 1.3	2.8 ±1.5
CRP (mg/L)	7.9 ± 9.3	0.2–39.0	8.9 ± 9.6	6.8 ± 9.0
ESR (mmhr)	16.5 ±16.6	1.0–77.0	18.0 ± 17.8	14.8 ± 15.2
Functional ability (HAQ-DI)	1.8 ± 0.6	1.0–3.0	1.7 ± 0.6	1.8 ± 0.6
Physical health (cardiovascular risk)				
Body mass index (kg/m^2^)	27.7 ± 4.9	19.4–42.0	27.5 ± 4.4	28.0 ± 5.5
Systolic blood pressure	136 ± 17	99–181	134 ± 17	139 ± 16
Diastolic blood pressure	82 ± 10	65–106	81 ± 10	83 ± 10
Cardiovascular risk score (QRISK2)	15.3 ±12.7	0.2–45.3	14.9 ± 13.8	15.8 ± 11.7
VO_2_ max (mL/min/kg)	20.6 ± 5.0	11.2–34.1	20.0 ± 5.1	21.4 ± 4.8

Included (*n* = 70) vs. excluded participants (*n* = 88) were not significantly different, in terms of age (*t* = 2.25, *p* = .64), gender, (*χ*^2^ (1) = 0.01, *p =* .94), disease duration (*t* = −0.15, *p* = .98), disease activity (DAS-28, *t* = −0.09, *p* = .13; CRP, *t* = 0.62, *p* = .56), functional ability (HAQ-DI, *t* = 0.96, *p =* .74), cardiorespiratory fitness (VO_2_ max, *t* = −0.79, *p* = .99), and overall cardiovascular risk (QRISK2, *t* = 1.14, *p* = .11), treatment with corticosteroids (*χ*^2^ (1) = 1.18, *p* = .18) and use of NSAIDS (*χ*^2^ (1) = 0.08, *p* = .78).

*DMARD* Disease Modifying Anti-Rheumatic Drug; *NSAID* Non-Steroidal Anti-Inflammatory Drugs; *HAQ-DI* Health Assessment Questionnaire-Disability Index.

#### Intention-to-treat

Of the *n* = 70 included participants at T1, *n* = 33 had missing data at T2 for at least one of the targeted variables (i.e., missing data; motivation regulations [*n* = 31], self-reported MVPA [*n* = 21], and subjective vitality [*n* = 33]). Questionnaire data were subsequently analyzed employing the principals of intention-to-treat. Specifically, single value imputation was employed, with missing data at 3 months (T2) imputed using participants’ baseline data (T1; i.e., last observation carried forward).

### Statistical analyses

Descriptive statistics were calculated and bivariate correlations computed to examine associations between targeted variables prior to multivariate path analyses. Conditions were coded as 0 = control and 1 = experimental (i.e., a positive correlation between the condition and autonomous motivation indicates a change in autonomous motivation in favor of the intervention). Path analysis with maximum likelihood estimation was employed in conjunction with bootstrapping to test the hypothesized model (Fig. 1b). Bootstrapping is a non-parametric, re-sampling procedure that does not impose the assumption of normality on the sampling distribution [38]. This approach is deemed superior to alternative tests in regards to Type-1 error rates and power, making it appropriate given the study sample size [38, 39].

Our hypothesized model stipulated direct paths between the condition at T1 and autonomous and controlled motivation at 3 months (end of the exercise program, T2). We also assumed direct associations between each composite motivation regulation at 3 months, with subjective vitality and MVPA at 3 months. For autonomous and controlled motivation, subjective vitality and MVPA at 3 months, a direct path is also stipulated from a variable that represents its corresponding baseline score (e.g., T1 autonomous motivation has a direct path to T2 autonomous motivation). Regressing T1 variables onto T2 variables in this way ensures that the T2 variable in the model represents a “change score” (e.g., change in the measured variable from baseline [T1] to the end of the exercise program [T2]).

Model fit was evaluated using the chi-square statistic (*χ*^2^), comparative fit index (CFI), and root square mean error of approximation (RMSEA, 90% CI, and [PCLOSE]). A non-significant *χ*^*2*^ (*p* = < .05), a CFI  >0.90, and an RMSEA of <0.08 specify a good fit between the model and data [40, 41]. For the RMSEA, a *p* of close fit [PCLOSE] statistic >0.05 also indicates a well-fitting model [42]. Where CFI is >0.95, and RMSEA is <0.06, the model is considered to demonstrate an excellent fit to the data [40]. The strength and direction of path coefficients were also considered in assessing the validity of the models. Standardized coefficients corresponding to 0.1, 0.3, and 0.5 were interpreted as small, medium, and large effect sizes, respectively [43].

Indirect effects were also determined via examination of the bootstrap bias-corrected 95% confidence intervals. The unique variance in levels of MVPA and subjective vitality at the end of the intervention that could be explained by a change in autonomous motivation (from T1 to T2) was determined via examining *R*^2^ values.

## RESULTS

Descriptive statistics are reported in Table 1. During the 3-month exercise program, *n* = 24 (75%) participants received at least 3 (maximum 4) consultations with the behavior change counselor, comprising at least (i) their baseline consultation (face-to-face), (ii) 1 × telephone consultation, and (iii) their exit consultation (face-to-face, 3 months). Overall, *n* = 29 (91%) of participants received at the minimum, both their initial (baseline) and exit (3 months) face-to-face consultations.

Table 2 reports sample means for autonomous and controlled motivation, self-reported MVPA, and subjective vitality at baseline (T1) and 3 months (T2). Cross-sectional and time-lagged associations between targeted variables are also indicated.

**Table 2:** Cross-sectional and time-lagged bivariate correlations between motivation regulations, self-reported MVPA, and subjective vitality

	Control	Experimental							
	*M* ± *SD*	*M* ± *SD*	1	2	3	4	5	6	7
Baseline (Time 1)									
1. Autonomous motivation	1.89 ± 0.70	2.05 ± 0.61							
2. Controlled motivation	1.22 ± 0.61	1.49 ± 0.62	.10						
3. MVPA (min/week)	761 ± 794	618 ± 647	−.08	−.08					
4. Subjective vitality	4.14 ± 1.34	4.21 ± 1.57	.24^†^	−.16	.07				
3 months (Time 2)									
5. Autonomous motivation	2.11 ± 0.81	2.80 ± 0.75	.51**	−.03	−.09	.37**			
6. Controlled motivation	1.05 ± 0.61	0.85 ± 0.68	.03	.60**	−.06	−.17	−.18		
7. MVPA (min/week)	699 ± 752	566 ± 577	−.00	−.02	.72**	.15	.10	−.02	
8. Subjective vitality	4.21 ± 1.39	4.56 ± 1.41	.25*	−.12	.12	.82**	.49**	−.20	.20

Cross-sectional associations are reported (T1 → T1), as well as time-lagged associations between variables measured at baseline and 3 months (T1 → T2).

*MVPA* moderate-to-vigorous physical activity.

*p < .05, **p < .01, ^†^*p =* .05.

Results revealed moderate levels of autonomous and controlled motivation at baseline for participants in both the experimental and control groups. The highest autonomous motivation and lowest controlled motivation were reported by participants in the experimental group at 3 months (T2). Participants in both groups self-reported engaging in MVPA above recommended levels (i.e., >150 min/week) at baseline and 3 months and demonstrated high levels of subjective vitality at both time points. Time-lagged correlation analyses indicated significant positive relationships between baseline (T1) and corresponding 3-month data (T2) for all variables. In addition, autonomous motivation at T1 was significantly and positively linked to subjective vitality at T2, with the same positive association observed between subjective vitality at T1 and autonomous motivation at T2. Controlled motivation at T1 and T2 was not linked to either MVPA or subjective vitality in the time-lagged analyses.

### Path analyses

**Fig 3:**
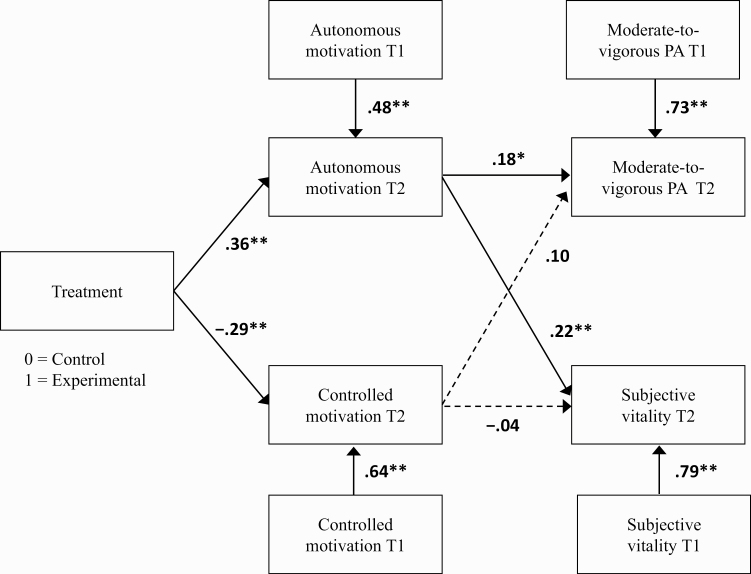
Data fit to the hypothesized model. Values represent path coefficients (β). *p < .05, **p < .01.

The hypothesized model demonstrated an excellent fit to the data (*χ*^2^ (26) = 28.69, *p* = .33, CFI = .99, RMSEA = 0.04 [90% CI: 0.00 to 0.11, PCLOSE = .55], Fig. 3). Results indicated that receiving the SDT-based intervention was associated with higher autonomous motivation and lower controlled motivation at the end of the 3-month exercise program (T2), after controlling for baseline (T1) values of these variables. In turn, autonomous motivation at 3 months significantly positively predicted self-reported MVPA and subjective vitality at 3 months. Analyses revealed autonomous motivation at 3 months to account for 3.1% and 2.7% of the unique variance in MVPA and subjective vitality, respectively. Controlled motivation did not significantly predict MVPA or subjective vitality.

### Indirect effects

Results demonstrated the intervention to have a significant indirect effect on subjective vitality (β = 0.08 [95% CI: 0.02 to 0.20]) at 3 months, via promoting more autonomous motivation toward PA, but not on MVPA (β = 0.04 [95% CI: −0.02 to 0.12]). No significant indirect effects were observed on MVPA and vitality via controlled motivation.

## DISCUSSION

The value of developing, delivering, and evaluating theory-based interventions to promote health behavior change is consistently underlined [13, 44]. The application of psychological theory—in this case, SDT—permits identification of intervention strategies to target determinants of the behavior (e.g., autonomy support) and specifies the hypothesized mechanisms or processes by which an intervention impacts intervention outcomes (e.g., autonomous motivation). In this study, we tested a theory-based process model of change to examine the effects of an SDT-based intervention on targeted psychological processes (i.e., autonomous motivation for PA) and related outcomes in RA. Results revealed autonomy-supportive PA counseling sessions (provided by a behavior change counselor) delivered in conjunction with an individually tailored RA exercise program to have a significant positive effect on participants’ autonomous motivation for PA. This positive change in autonomous motivation, in turn, significantly predicted higher levels of MVPA and improved feelings of subjective vitality. As such, results suggest a change in autonomous motivation is the psychological mechanism (“process of change”) underlying the positive changes in MVPA and subjective vitality observed among RA patients in this intervention.

To date, several existing interventions have been developed and tested, with the aim of supporting PA behavior change in RA. It should be noted, however, that these interventions have largely not specified a theoretical basis [45] or have only loosely referred to a theoretical framework in outlining the design and evaluation of their intervention. Overall, these interventions have demonstrated variable success in promoting PA, but due to their lack of theoretical-underpinning, it is not clear what were the psychological mechanisms that may have accounted for any observed changes in PA behavior following the intervention. In this SDT-based intervention, we specified autonomous motivation for PA as the determinant (a psychological mechanism) targeted by the intervention. In testing the theoretical process model (Fig. 1b), we demonstrated that autonomy-supportive interactions promoted more autonomous motivation for PA and, in turn, encouraged higher levels of engagement in MVPA and more optimal psychological functioning among RA patients.

Present results hold implications for the manner in which discussions regarding PA are delivered to people living with RA within the clinical context. Specifically, findings suggest that supplementing RA exercise programs with autonomy-supportive interactions may promote more autonomous reasons for engaging in PA and thus foster internalization and positive PA behavior change. In this intervention, these autonomy-supportive exchanges may have inspired RA patients receiving the intervention to experience greater volition and self-endorsement of their actions and, consequently, increase their engagement in health-enhancing PA (i.e., MVPA). As such, results highlight (i) the potential value of autonomy-supportive interactions as intervention strategies to promote PA in RA and (ii) the role of autonomous motivation as a modifiable determinant of PA in RA, while also demonstrating support for the theoretical tenets of SDT.

While there was no significant association observed between changes in controlled motivation with changes in MVPA in this study, a small, weak positive relationship between these variables was reported. This may be a result of the fact that study participants in both control and experimental arms were aware that they had consented to take part in an intervention with the aim of encouraging exercise. As a result, they felt some expectations both internally (introjected regulation) and externally (external regulation) to engage in MVPA. It is possible that the sample size in this study did not allow a significant association between controlled motivation and MVPA to be detected. Thus, future research with larger samples (and greater statistical power) is required to explore the role of controlled motivation for engagement in PA among people living with RA. However, it is also important to recognize that while these results may provide initial evidence to suggest that controlled motivation may serve to encourage MVPA adoption among people living with RA, it is unlikely to foster the maintenance of PA in the longer term [15, 17]. Indeed, studies show that controlled motivation to engage in PA is not linked to PA maintenance in intervention studies with at least 6 months follow-up, and recommendations are therefore centered around promoting autonomous motivation (and minimizing controlled regulation) for PA engagement [18].

Ours is the first theory-based PA intervention to examine and demonstrate a significant effect of autonomy-supportive interactions on psychological well-being in RA, through enhancing autonomous motivation. Results suggested that RA patients who demonstrated greater internalization of their reasons for engaging in PA over the 3-month intervention (i.e., experienced enhanced autonomous motivation for PA) also reported improved feelings of vitality. It is possible that the autonomy-supportive interactions with the behavior change counselor inspired RA patients to feel more “in control” (i.e., acting of their own volition) and better equipped psychologically to cope with the physical challenges resulting from RA, through engaging in PA [26, 28]. Thus, findings may suggest interventions targeting autonomous motivation for PA might not only to translate to positive changes in PA, but also to RA patients experiencing more optimal psychological well-being.

Similar findings to those observed herein have been reported where SDT-based PA interventions have been delivered in other health care settings (e.g., primary care, exercise referral) [17]. Thus, the provision of autonomy-supportive strategies—delivered by key health care professionals—may offer an effective PA intervention strategy across the broader clinical context [46]. Indeed, health care professionals are important resources for promoting PA due to their potential to reach a considerable number of patients and their credibility from the patient perspective. However, the health care context presents unique challenges, such as constraints on time and resources. Consequently, it is critical that pathways to implementation are developed that consider these challenges in parallel to the delivery of PA promotion programs. The World Health Organisation has proposed that successful PA implementation can be achieved with the capacity building of frontline health care practitioners managing the patients (e.g., rheumatologists) [47]. Inferring from this study, such capacity building would constitute conveying information about PA and its health benefits in RA, as well as instruction in the delivery of intervention approaches to support autonomous motivation for PA. However, to ensure consistency and confidence in PA messaging, such capacity building is likely to require substantial training provisions for rheumatology health professionals, integrated at all stages of their career. This will likely come at a considerable cost and relies on commitment from both Health Service systems and educational partners. Thus, an alternative approach to implementation may be to integrate specialized PA behavior change professionals into Rheumatology care pathways, with expertise in relevant behavioral theories (in this case, SDT), who are trained in strategies to promote autonomous motivation for PA.

While significant effects were observed for key outcomes tested in our SDT-based process model of change, challenges in implementation and measurement may have influenced intervention efficacy and fidelity. First, we were not able to administer an objective measure to evaluate the autonomy-supportive interactions (either face-to-face or via telephone), to ascertain the degree to which intervention strategies were delivered as specified and in accordance with the principles of SDT [48]. Second, while all participants received their initial face-to-face consultation at the commencement of the RA-tailored exercise program, not all participants received follow-up telephone calls and participated in their exit consultation. This resulted in some participants receiving a lower “dose” of intervention than anticipated and may have impacted present results.

Nevertheless, positive changes in autonomous motivation for PA and MVPA were observed, where at least one SDT-based, face-to-face consultation was received upon beginning the exercise program. Thus, at the minimum, the initial exchanges between the behavior change counselor and patients may have been sufficient to promote internalization of reasons for PA engagement and inspire positive PA behavior change. In this regard, it would be interesting for future intervention studies to elucidate the minimally important “dose” of intervention required (both number and type of SDT-based exchanges) to observe positive (and clinically meaningful) changes in PA behavior among people with RA. This will have implications for facilitating the implementation of PA advice in this population, enabling efficiency in terms of time and resources. In this study, 91% (*n* = 29/32) of participants received both their initial (baseline) and exit (3 months) face-to-face consultations with the behavior change counselor, and 75% (*n* = 24/32) of participants received both their face-to-face consultations and at least one follow-up telephone call (at 1 or 2 months). Thus, it is likely that two to three SDT-based consultations may be required to encourage the initial adoption of PA among this patient group.

In interpreting the results of the current study, the discrepancy between the relationships observed in our hypothesized model versus changes in pre- and post-intervention levels of MVPA should also be acknowledged. In Table 2, minutes of MVPA decreased between baseline and the end of the exercise intervention in both the experimental and control groups. According to our hypothesized model, we would expect increased estimates of MVPA among participants who received the SDT-based intervention. That is, the more self-determined one is (i.e., more autonomous motivation for PA) the higher their engagement in MVPA. The overall decreases in MVPA observed at the group level among participants in the experimental arm can therefore not be explained by the relationships specified in the hypothesized model. The group-level decrease in MVPA may therefore result from other factors that we were not able to measure in our study (e.g., environmental factors such as weather) and/or account for in our statistical analysis (e.g., disease activity). In addition, it is possible that after completing the RA-tailored exercise program (which both groups received), the participants in both arms changed how they reported their MVPA (e.g., due to increased awareness of what constituted MVPA), accounting for the decrease seen in both the experimental and control groups. Further, a reliance on self-reported MVPA may have also affected the validity of MVPA estimates in this study. While we determined MVPA assessed via an accelerometer in the larger protocol [29], compliance with device wear at 3 months (T2) was not sufficient to permit evaluation of the theory-based process model using accelerometer-assessed MVPA. Future research employing device-based assessments of PA (e.g., accelerometers) is warranted to corroborate the value of SDT-based interventions for promoting uptake (and subsequent maintenance) of MVPA among people living with RA.

The generalizability of current results also warrants further discussion. Participants recruited to this study represented a cohort of RA patients with controlled diseases activity (DAS-28 = <3.2) and who were willing to undertake a lifestyle change in regards to their PA. Moreover, levels of self-reported MVPA by both groups, at all time points, were higher than the recommended 150 min/week, and subjective vitality scores indicated that participants in general reported positive mental health. It is therefore possible that findings may not be generalizable to RA patients with active disease, who are considered “physically inactive” (according to the 150 min/week MVPA criteria), with more compromised psychological well-being, and/or those who are not yet ready to engage in behavioral change. The efficacy of this SDT-based exercise intervention should therefore be tested among a more heterogeneous sample of RA patients, as intervention approaches and strategies may need to be adapted to consider these factors. With that said, the high levels of MVPA observed in this study may to some extent represent a mismatch between the manner in which government recommendations for PA are framed (i.e., largely based on self-report of structured exercise and purposeful MVPA) and the questions asked in the IPAQ: that is, the IPAQ assessing MVPA accumulated during all lifestyle activities, across several life domains (e.g., occupational, domestic, and leisure time MVPA) [49]. As such, it is perhaps not surprising that levels of MVPA are reported to exceed 150 min/week in this study. Indeed, it is likely that levels of MVPA among the current sample of people with RA are lower than those reported herein.

It is also important to recognize that the missing data at T2, and the manner in which these data were imputed, may have impacted the results reported. Single imputation was used to compute missing data in this study. This method does not account for the uncertainty of missing data and may result in standard errors of the estimates that are likely too small, potentially leading to Type-1 error [50]. The results of this study should therefore be interpreted considering these limitations, as the precision of the results and variability in MVPA and subjective vitality explained by our hypothesized model may have been overestimated. Still, even in acknowledging these limitations, the results of this study add an important contribution to the literature. The findings provide novel evidence of the potential value of SDT-based interventions for promoting MVPA and vitality among people with RA and as a result, provide an informed basis upon which to develop future interventional efforts. Future work in this area should aim to confirm the value of autonomy-supportive strategies for encouraging MVPA and increasing feelings of vitality in RA.

## CONCLUSIONS

This is the first RCT to investigate whether SDT-based PA consultations delivered in conjunction with an RA-tailored exercise program were effective for promoting autonomous motivation for PA among people with RA. Overall, the results of the trial are in line with the motivational processes hypothesized by SDT. That is, results demonstrated that autonomy-supportive PA consultations correspond to positive changes in participant’s autonomous motivation for PA and, subsequently, their engagement in (self-reported) MVPA and subjective vitality on the conclusion of a 3-month RA exercise program. As such, interventions targeting autonomous motivation as a key psychological determinant of PA behavior in RA may provide a valuable contribution to encouraging PA behavior change in these patients. The provision of autonomy-supportive strategies targeting PA may offer an effective PA intervention strategy in this regard.
